# DPP3/CDK1 contributes to the progression of colorectal cancer through regulating cell proliferation, cell apoptosis, and cell migration

**DOI:** 10.1038/s41419-021-03796-4

**Published:** 2021-05-22

**Authors:** Yixin Tong, Yuan Huang, Yuchao Zhang, Xiangtai Zeng, Mei Yan, Zhongsheng Xia, Dongming Lai

**Affiliations:** 1 Department of Tongji Medical College of Huazhong University, 1095 Jiefang Dadao, Wuhan, Hubei Province China; 2 Department of Endoscopy Center, Zhongshan Hospital of Fudan University, 180 Fenglin Road, Xuhui, Shanghai China; 3 Department of Gastrointestinal Surgery, Sun Yat-sen memorial Hospital of Sun Yat-sen University, 107 Yanjiang West Road, Guangzhou, Guangdong Province China; 4 Department of The First Affiliated Hospital, Gannan Medical University, 23 Qingnian Road, Zhanggong District, Ganzhou, Jiangxi Province China

**Keywords:** Cancer, Diseases

## Abstract

At present, colorectal cancer (CRC) has become a serious threat to human health in the world. Dipeptidyl peptidase 3 (DPP3) is a zinc-dependent hydrolase that may be involved in several physiological processes. However, whether DPP3 affects the development and progression of CRC remains a mystery. This study is the first to demonstrate the role of DPP3 in CRC. Firstly, the results of immunohistochemistry analysis showed the upregulation of DPP3 in CRC tissues compared with normal tissues, which is statistically analyzed to be positively correlated with lymphatic metastasis, pathological stage, positive number of lymph nodes. Moreover, the high expression of DPP3 predicts poor prognosis in CRC patients. In addition, the results of cell dysfunction experiments clarified that the downregulation of DPP3 significantly inhibited cell proliferation, colony formation, cell migration, and promoted apoptosis in vitro. DPP3 depletion could induce cell apoptosis by upregulating the expression of BID, BIM, Caspase3, Caspase8, HSP60, p21, p27, p53, and SMAC. In addition, downregulation of DPP3 can reduce tumorigenicity of CRC cells in vivo. Furthermore, CDK1 is determined to be a downstream target of DPP3-mediated regulation of CRC by RNA-seq, qPCR, and WB. The interaction between DPP3 and CDK1 shows mutual regulation. Specifically, downregulation of DPP3 can accentuate the effects of CDK1 knockdown on the function of CRC cells. Overexpression of CDK1 alleviates the inhibitory effects of DPP3 knockdown in CRC cells. In summary, DPP3 has oncogene-like functions in the development and progression of CRC by targeting CDK1, which may be an effective molecular target for the prognosis and treatment of CRC.

## Introduction

Colorectal cancer (CRC) is the result of the gradual accumulation of a series of genetic and epigenetic changes in normal colonic epithelial cells, contributing to colorectal adenomas and invasive adenocarcinomas^1^. It is one of the leading causes of cancer-related death worldwide and has the characteristics of high incidence^2^. The latest statistics show that the mortality and morbidity of the population are rising every year^2^. Although surgery, radiotherapy, and chemotherapy have been developed, the clinical prognosis is still not ideal, especially for CRC patients with metastasis^3^. In addition, first-line drugs for adjuvant therapy have been used in patients with CRC, such as irinotecan, 5-fluorouracil, and its derivatives, platinum drugs^4–6^. Unfortunately, the heterogeneity of tumors leads to serious consequences of chemotherapy and drug resistance^7,8^. In general, the clinical efficacy and prognosis are terrible as well as drug resistance is serious for CRC patients. Recently, potential therapeutic methods such as molecular targeted therapy have become a hot spot^9,10^. Therefore, the study of the molecular mechanism of CRC lays the foundation for the development of molecular targeted therapy, which is of great significance and has attracted considerable attention.

Exopeptidase dipeptidyl peptidase 3 (DPP3) is a member of the zinc-dependent M49 metallopeptidase family that cleaves dipeptides at the N-terminal site^11,12^. Human DPP3, a 29,647 bp gene (NC_000011.10), located on chromosome 11q12-13.1^13^. The primary biological function of DPP3 is to regulate the transformation of enkephalin, opioid peptide, terminal protein, and cell signaling^11^. Previous studies indicated the involvement of DPP3 in cancer. For example, DPP3 is overexpressed in ovarian carcinoma, endometrial cancers, lung squamous cell carcinoma, and glioblastoma cells^14–16^. In addition, Simaga et al. supported that increased DPP3 activity is associated with histologic aggressiveness of cancer^17^. However, little information is available on the expression, specificity, and function of DPP3 in human cancers.

In this study, we determined the cellular function and molecular mechanisms of DPP3 in CRC. Firstly, the differential expression of DPP3 in CRC and adjacent normal tissues was observed by immunohistochemically staining. Subsequently, the correlation between DPP3 expression and tumor characteristics was analyzed, revealing the role of DPP3 in the progression and prognosis of CRC patients. Furthermore, DPP3 was downregulated by lentivirus expression of DPP3-targeted shRNA in CRC cells to investigate its regulation of cell proliferation, apoptosis, and migration. In addition, the potential molecular mechanism of DPP3 promoting CRC was further explored by RNA sequencing and verified by compensation experiments. All in all, this study is the first attempt to interpret the potential carcinogenic activity of DPP3 in CRC.

## Materials and methods

### Clinical tissues microarray and immunohistochemically (IHC) staining

The paraffin-embedded human CRC chips were purchased from Shanghai Outdo Biotechnology Co., Ltd. (# HCoLA180SU15), which included 99 cases of cancerous tissue and 76 cases of normal tissue. Meanwhile, the detailed pathological characteristics of clinical specimens were obtained, and all patients signed the informed consent. The sample inclusion criteria were as follows^1^: The patient was pathologically diagnosed with CRC prior to surgery^2^; The patient had no primary malignancy of other organs prior to diagnosis of CRC^3^; The patient did not receive radiotherapy and chemotherapy. The Ethics Committees of the Sun Yat-sen memorial Hospital of Sun Yat-sen University have approved all studies about human participants. All participants have written informed consent.

After the tissue sections were dewaxed, rehydrated, and blocked, the primary antibody (Table S1) was added and incubated at 4 °C overnight. After they were washed with phosphate buffer saline (PBS), the goat anti-rabbit IgG polyclonal antibody labeled with horseradish peroxidase (HRP) was incubated at room temperature for 30 min. DAB and hematoxylin were used for staining tissue sections. The immunohistochemical scores of the specimens were determined according to the sum of staining intensity and staining degree. The low or high expression parameters of DPP3 were determined by the median of IHC score of all tissues. Those lower than the median were regarded as low expression, and vice versa.

### Cell culture

Human CRC cell lines, DLD-1, SW480, HCT 116, and RKO were purchased from Cell Bank of Chinese Academy of Sciences (Shanghai, China) and cultured in a humidified incubator at 37 °C with 5% CO_2_. In detail, DLD-1 and SW480 were maintained in 90% DMEM-H supplemented with 10% FBS. HCT 116 cells were cultured in 90% RPMI 1640 (Corning) supplemented with 10% fetal bovine serum (FBS). RKO cells were cultured in 90% EMEM with 10% FBS. HCT 116 and RKO cells were recently authenticated by STR profiling (Supplementary materials) and tested for mycoplasma contamination.

### Target gene knockdown cell models

RNA interference target sequences were designed using human DPP3 and CDK1 genes as templates (detailed in Table S2). Subsequently, RNA interference sequences targeting DPP3 and CDK1 were cloned into LV-004/BR-V-108 vectors, respectively, and lentivirus vectors were constructed after packaging (Shanghai Bioscienceres, Co., Ltd.). Afterward, 400 μL recombinant lentivirus vectors (1 × 10^7^ TU/well) were infected with HCT 116 and RKO cells by Lipofectamine 2000 (Invitrogen). After 72 h, the cell fluorescence was observed by fluorescence microscope (OLYMPUS). The knockdown efficiency of DPP3 and CDK1 was verified by quantitative PCR (qPCR) and western blot (WB). Notably, the shCtrl group as negative control; shDPP3 and shCDK1 was the cells that knockdown of DPP3 or CDK1, respectively; shCDK1 + shDPP3 was the cells that knockdown of CDK1 and DPP3 simultaneously; NC(OE + KD) was normal cells infected with empty vector (LV-004 and BR-V108), as negative control; CDK1 + NC-shDPP3 was normal cells infected with CDK1 and NC-shDPP3 lentivirus for overexpressing CDK1; shDPP3 + NC-CDK1 was normal cells infected with shDPP3 and NC-CDK1 lentivirus for downregulating DPP3; CDK1 + shDPP3 was cells with simultaneously downregulating DPP3 and overexpressing CDK1.

### qPCR

The total RNA in CRC cells was extracted with Trizol reagent and then reversed transcription to obtained cDNA using Promega M-MLV Kit (Promega Corporation). The RNA level was detected by the qPCR detection system, and the relative quantitative analysis of RNA was calculated by 2^−∆∆CT^ method. Primer sequences in this experiment were listed in Table S2 and GAPDH was an internal control.

### Western blot (WB)

HCT 116 and RKO cells were collected and lysed by Lysis Buffer. After protein concentration was detected through BCA Protein Assay Kit (HyClone-Pierce), and then 20 μg proteins were subjected by 10% SDS-PAGE. Subsequently, they were transferred to polyvinylidene difluoride (PVDF) membranes and blocked with TBST containing 5% skim milk at 4 °C for 1 h. After that, they were first incubated with primary antibodies (listed in Table S1) overnight at 4 °C and then with Horseradish peroxidase (HRP) conjugated IgG polyclonal antibodies for 2 h at room temperature. Finally, ECL plusTM WB system kit was performed to color develop and detect.

### Co-immunoprecipitation (Co-IP) assay

In terms of Co-IP analysis, 20 μL protein Amax G beads were added to the 1.2 mg protein lysate and incubated at 4 °C for 2 h, then continued to incubate overnight with the required antibodies (Table S1). The beads were collected after centrifugation (2000 × g), the appropriate IP lysate and 5 × loading buffer were added and maintained at 100 °C for 10 min. After HCT 116 samples were separated by 10% SDS-PAGE and the next steps were performed as described in the WB.

### MTT assay

HCT 116 and RKO cells were suspended and transferred to the 96-well plate (2000 cell/well). MTT (5 mg/mL; Genview) 20 μL were added to each well for 4 h, and then 100 μL dimethyl sulfoxide (DMSO) was added for 5 min. Finally, the absorbance was detected at 490 nm and 570 nm wavelengths using a microplate reader (Tecan infinite).

### Celigo cell counting assay

After HCT 116 cells were inoculated into 96-well plates, 1640 medium was added and supplemented with 10% FBS for further culture for 5 days. At the same time, the medium was changed every 3 days. Following, cell counts were performed using Celigo image cytometer on days 1, 2, 3, 4, and 5 and the data were analyzed.

### Colony formation assay

HCT 116 cells were suspended and seeded into 6-well plates with 500 cells/well. After a week of continuous culture, the cells were immobilized with 4% paraformaldehyde and then stained with GIEMSA (butyl fruit biotechnology) and photographed with a digital camera. The proliferative potential of individual cells was assessed by counting the clone formation rate.

### Fluorescence activated cells sorting (FACS)

The apoptosis rate and cycle distribution of HCT 116 and RKO cells were determined by FACS. When the fusion degree reached 80%, the cells were harvested, centrifuged (1300 rpm) for 5 min and washed with 4 °C precooled D-Hanks. The cells were re-suspended with 200 μL binding buffer and incubated with 10 μL Annexin V-APC for 15 min avoid light. Cell apoptosis rate was calculated in three randomly selected visual fields.

HCT 116 and RKO cells were harvested, centrifuged (1300 rpm), and suspended. Afterward, they were washed with PBS and labeled with 500 μL PI (BD Biosciences, Franklin Lakes, NJ, USA). The FACSCanto II Flow Cytometry was used to analyze the ratio of cells in the G1, S, and G2 phases distribution.

### Human apoptosis antibody array

The differential expression of related proteins in the apoptosis signaling pathway was detected by human apoptotic antibody array after DPP3 was downregulated in HCT 116 cells. The cells proteins were incubated with blocked antibody array membrane overnight at 4 °C. After washing, Detection Antibody Cocktail (1:100) was incubated for 1 h, followed by with HRP linked streptavidin conjugate for 1 h. All spots were visualized by enhanced ECL and the signal densities were analyzed with Image J software (National Institute of Health).

### Transwell assay

HCT 116 and RKO cells were digested with trypsin and suspended in a low serum medium. It was removed from the Transwell room and washed with PBS. The methanol was fixed for 30 min and 0.1% crystal violet was stained for 20 min. Finally, the cells are viewed under microscope, photographed, and counted.

### Wound-healing assay

HCT 116 and RKO cells (5 × 10^4^ cells/well) were inoculated into a 96-well dish for culturing until cell confluence reached 90% and scratched on the cell layer using 96 wounding replicators (VP scientific). At the same time, the medium was substituted with the 1% FBS contained fresh medium. Photographs of the wound were captured at pre-set time points (0 h, 24 h, and 48 h). Finally, the percentage of migration was evaluated utilizing Image Pro Plus.

### Tumor-bearing animal model

Animal experiments were approved by the Ethics Committee of Sun Yat-sen memorial Hospital of Sun Yat-sen University and conducted in accordance with guidelines and protocols for animal care and protection. The 4-week-old female BALB/c nude mice (*n* = 20) were purchased from Shanghai Lingchang Experimental Animal Co., Ltd., and were randomly divided into shCtrl (*n* = 10) and shDPP3 (*n* = 10) groups with 10 of each group. RKO cells infected with shRNA lentivirus were suspended and 200 μL cell suspensions (4 × 10^6^ cells) were subcutaneously injected into the right forelimb of each mouse. After 5 days of injection, data were collected 2–3 times a week, including weighing animals, measuring tumor length, and short diameter. The mice were continuously fed for 25 days, which were intraperitoneally injected with 0.7% sodium pentobarbital (10 μL/g) for anesthesia and subsequently placed under IVIS Spectrum (Perkin Elmer) for imaging and fluorescence observation. Subsequently, the mice were sacrificed and the tumors were excised to measure the volume and weight of the tumors. Finally, IHC staining of tumor tissues in mice to determine Ki67 expression levels.

### Prime view human gene expression array

The total RNA of HCT 116 cells was obtained by TRIZOL reagent, whose concentration and integrity of RNA were tested by Agilent bio analyzer 2100. The qualified total RNA was further purified by RNeasy mini kit (#74106, QIAGEN) and RNase-Free DNase Set (#79254, QIAGEN). Following the manufacturer’s instruction of Affymetrix human GeneChip 3′ IVT PLUS Reagent Kit (#902416) to obtain biotin labeled cRNA. Moreover, array hybridization and wash were completed using GeneChip® Hybridization, Wash and Stain Kit (#900720, Affymetrix). Finally, slides were scanned with GeneChip® Scanner 3000 (#00-00212) and Command Console Software 4.0.

### Statistical analysis

All the experiments in this study were performed in triplicate, and the data were expressed as the mean ± standard deviation. Student’s *t* Test was used to analyze the statistical significance between two groups and one-way ANOVA test was used for multi-groups. All statistical analysis was carried out by GraphPad Prism software and *P* < 0.05 was considered statistically significant.

## Results

### The high expression of DPP3 in CRC is significantly correlated with poor prognosis

**Fig. 1:**
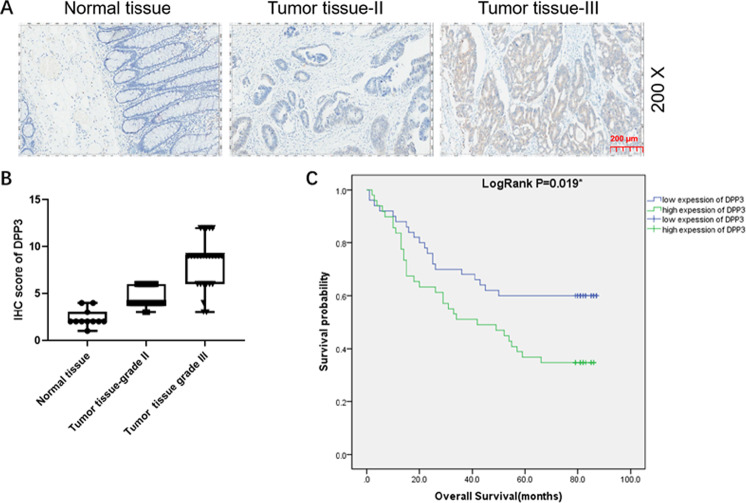
DPP3 was upregulated in CRC and knockdown of DPP3 cells models was constructed. **A**, **B** The expression level of DPP3 was detected by IHC analysis in normal tissues and CRC tissues with different T stage. **C** Kaplan–Meier survival analysis was performed to establish the relationship between DPP3 expression and prognosis of CRC patients. The representative images were selected from at least three independent experiments. Data were shown as mean ± SD. *P < 0.05, **P < 0.01, ***P < 0.001.

To preliminarily clarify the role of DPP3 in CRC, the difference in expression of DPP3 in tumor tissues and normal tissues was observed through IHC. The representative pictures of IHC staining were illustrated in the Fig. 1A, indicating that expression of DPP3 was upregulated in CRC. The statistical analysis of expression data from 99 CRC and 76 normal tissues showed that DPP3 expression was generally higher in the CRC (*P* < 0.001, Table 1, Fig. 1B). Furthermore, the results of Mann–Whitney *U* analysis showed that the expression of DPP3 was positively correlated with lymphatic metastasis (*N* value), (*P* = 0.034), pathological stage (*P* = 0.008), positive number of lymph nodes (*P* = 0.012) and other pathological data (Table 2). This result was further verified by Spearman rank correlation analysis, indicating that the increased expression of DPP3 is a warning of deterioration in patients with CRC (Table 3). In addition, according to Kaplan–Meier survival analysis, the high expression of DPP3 in tumors predicted the overall survival time was relatively short (Fig. 1C). Comprehensive analysis suggested that the expression level of DPP3 possessed clinical value in predicting the poor prognosis of CRC patients.

**Table 1:** Expression patterns in colorectal cancer tissues and para-carcinoma tissues revealed in immunohistochemistry analysis.

DPP3 expression	Tumor tissue		Para-carcinoma tissue		*P* value
	Cases	Percentage	Cases	Percentage	
Low	50	50.5%	76	100%	<0.0001
High	49	49.5%	0	–

**Table 2:** Relationship between DPP3 expression and tumor characteristics in patients with colorectal cancer.

Features	No. of patients	DPP3 expression		*P* value
		Low	High	
All patients	99	50	49	
Age (years)				0.159
<69	49	28	21	
≥69	49	21	28	
Gender				0.839
Male	57	28	29	
Female	41	21	20	
Grade				0.462
II	72	38	34	
III	27	12	15	
T Infiltrate				0.312
T2	4	1	3	
T3	63	31	32	
T4	32	18	14	
Lymphatic metastasis (*N*)				0.034
N0	51	32	19	
N1	36	12	24	
N2	12	6	6	
Stage				0.008
1	4	1	3	
2	46	31	15	
3	44	18	26	
4	5	0	5	
Tumor size				0.685
≤5 cm	56	29	27	
>5 cm	42	20	22	
Lymph nodes				0.272
≤6	50	28	22	
>6	49	22	27	
Lymph node positive				0.012
≤0	51	32	19	
>0	48	18	30

**Table 3:** Relationship between DPP3 expression and tumor characteristics in patients with colorectal cancer.

Features		*P* value
stage	Pearson correlation	0.267
	Significance (double-tail)	0.008
	*N*	99
lymphatic metastasis (*N*)	Pearson correlation	0.214
	Significance (double-tail)	0.033
	*N*	99
Lymph node positive	Pearson correlation	0.252
	Significance (double-tail)	0.012
	*N*	99

### Construction of DPP3 knockdown in CRC cells

As expected, qPCR detection showed highly abundant expression of DPP3 in CRC cell lines, including DLD-1, RKO, HCT116, and SW480 (Fig. S1A), among which HCT 116 and RKO were chosen for subsequent investigations. Moreover, the knockdown efficiency of three shRNA silencing DPP3 was screened by qPCR, among which shDPP3 (RNAi-15671) and shDPP3 (RNAi-15672) were used in the follow-up experiments (Fig. S1B). Furthermore, green fluorescent signal was observed in 80% of the cells indicating successful transfection (Fig. S1C). Moreover, the downregulation of DPP3 in mRNA and protein levels was proved by qPCR (Fig. 1D) and WB (Fig. 1E), respectively, confirmed the successful knockdown of DPP3 in HCT 116 and RKO cells. Similarly, we stably knocked down DPP3 in HCT 116 and RKO cells using another shDPP3 target (Fig. S2A–C). Subsequently, the cell lines were subjected to loss-of-function assays to investigate the effects of altered DPP3 expression.

### Depletion of DPP3 inhibits the malignant biological behavior of CRC in vitro

**Fig. 2:**
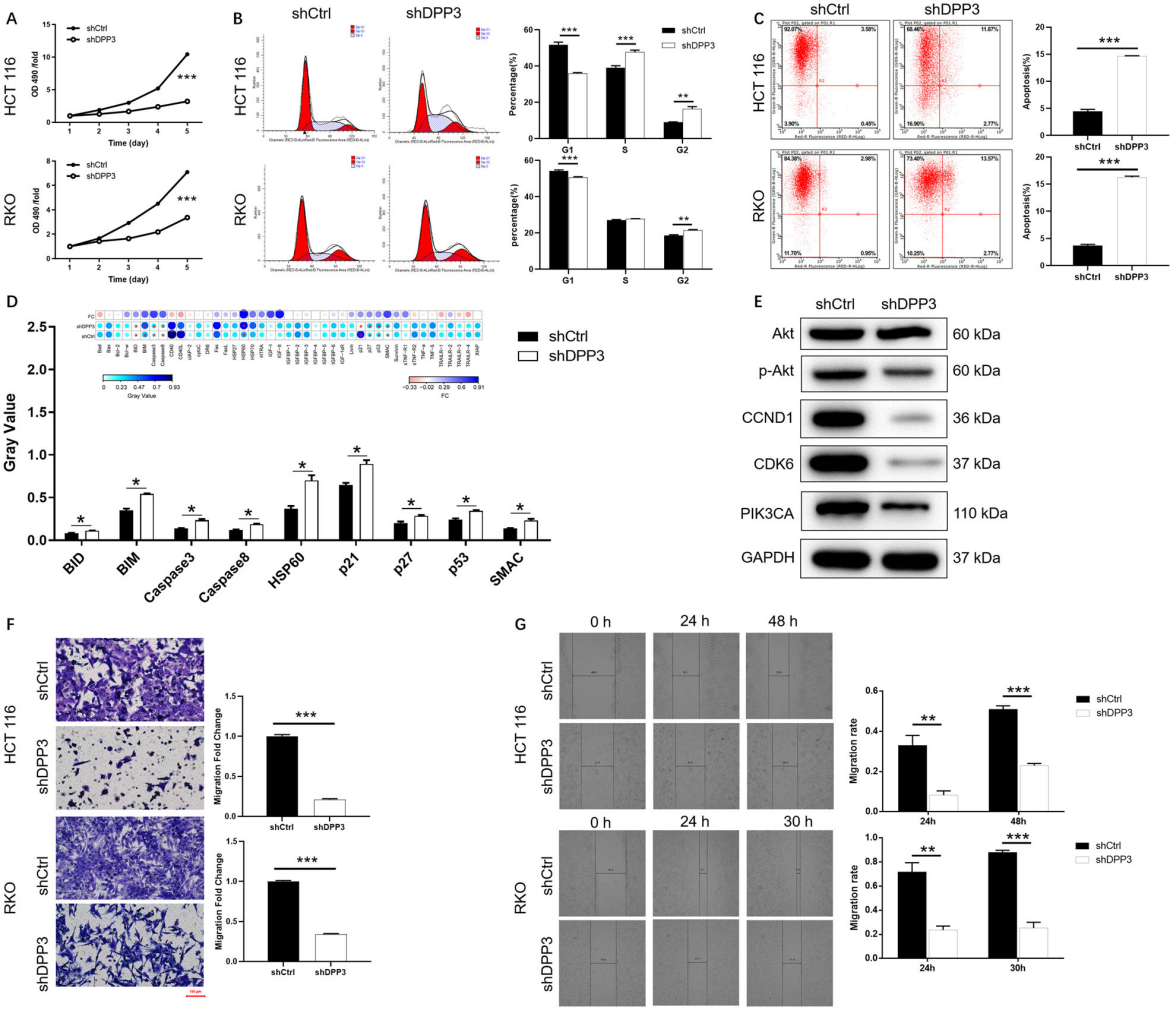
DPP3 knockdown inhibited CRC development in vitro. **A** MTT assay was employed to show the effects of DPP3 on cell proliferation of RKO and HCT 116 cells. **B**, **C** Flow cytometry was performed to detect cell cycle (**B**) and cell apoptosis (**C**) of RKO and HCT 116 cells with or without DPP3 knockdown. **D** Human apoptosis antibody array was utilized to analyze the regulatory ability of DPP3 on expression of apoptosis-related proteins in HCT 116 cells. **E** The protein expression of downstream molecular in HCT 116 cells with or without DPP3 knockdown was measured by WB. **F**, **G** CRC cell migration ability was accessed by Transwell assay (**F**) and wound-healing assay (**G**). The representative images were selected from at least three independent experiments. Data were shown as mean ± SD. *P < 0.05, **P < 0.01, ***P < 0.001.

**Fig. 3:**
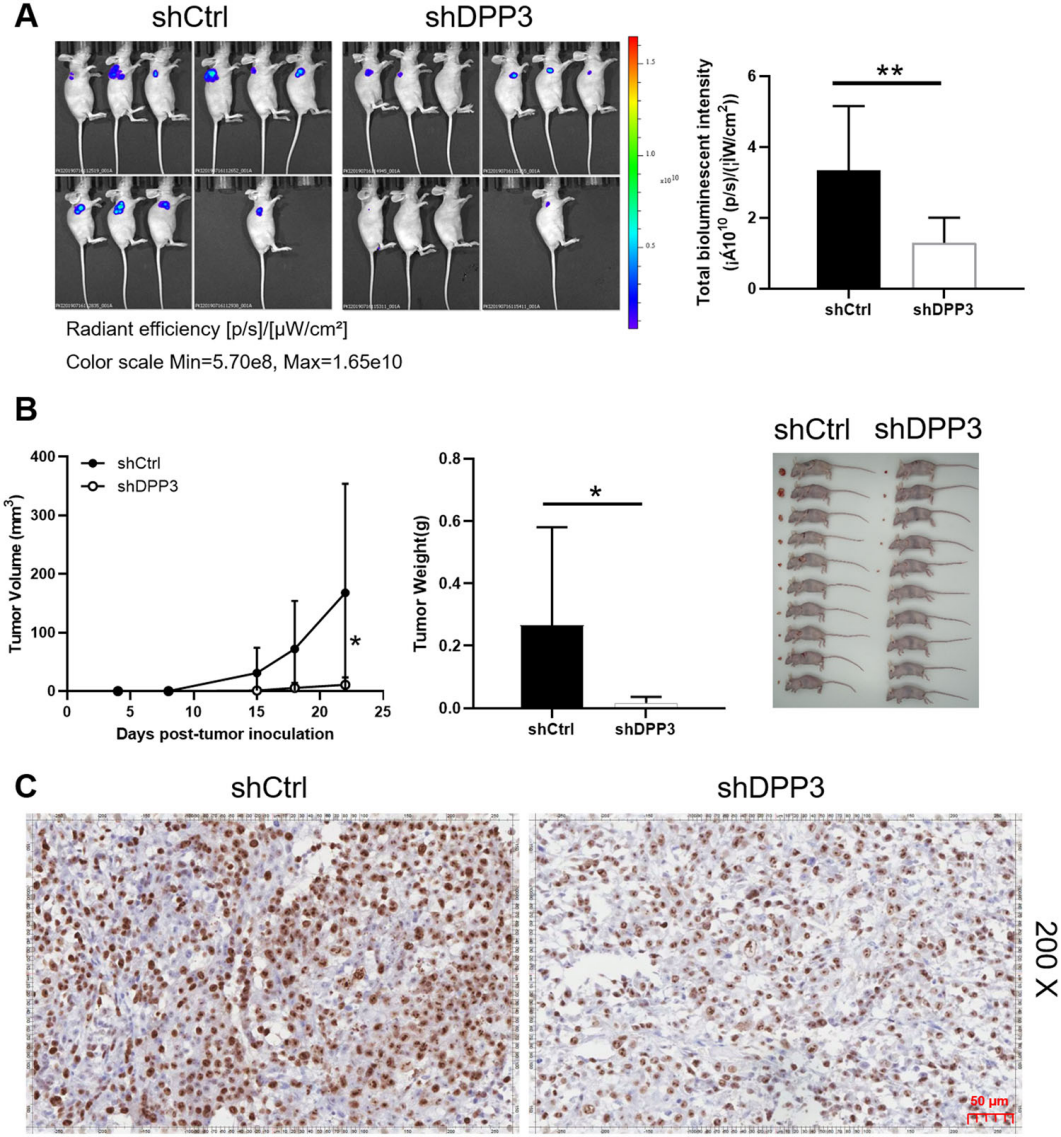
DPP3 knockdown inhibited CRC development in vivo. **A** In vivo imaging was performed to evaluate the tumor burden in mice of shDPP3 and shCtrl groups post tumor-inoculation; The bioluminescence intensity was scanned and used as a representation of tumor burden in mice of shDPP3 and shCtrl groups. **B** Inset: photo of the removed tumors was taken post tumor-inoculation; RKO cells with or without DPP3 knockdown, the volume of tumors formed in mice was measured and calculated at indicated time intervals; Mice were sacrificed and the tumors were removed for weighing. **C** The Ki67 level in tumors removed from mice was detected by IHC as a representation of tumor growth. Data were shown as mean ± SD. *P < 0.05, **P < 0.01, ***P < 0.001.

For the sake of verifying the function of DPP3 in CRC cells, we explored a range of biological processes, including proliferation, apoptosis, cycle, and migration ability. First, HCT 116 and RKO cells with DPP3 deletion (shDPP3) grow much more slowly than that without deletion of DPP3 (shCtrl) from results of MTT (*P* < 0.001, Fig. 2A). Besides, cell cycle and apoptosis of CRC cells with or without DPP3 knockdown were evaluated through flow cytometry. Compared with the control group, HCT 116 and RKO cells downregulated by DPP3 were arrested in G2 phase (*P* < 0.001, Fig. 2B). Meanwhile, cells deficient in DPP3 showed more apoptotic cell populations than those in shCtrl group (*P* < 0.001, Fig. 2C). The results of human apoptotic antibody array indicated that DPP3 depletion could upregulate the expression of BID, BIM, Caspase3, Caspase8, HSP60, p21, p27, p53, and SMAC in HCT 116 (*P* < 0.05, Fig. 2D). On the other hand, knockdown of DPP3 could result in downregulation of p-Akt, CCND1, CDK6, and PIK3CA were downregulated (Fig. 2E). In addition, migration ability of cells with or without DPP3 knockdown was investigated by Transwell (*P* < 0.001, Fig. 2F) and wound-healing assays (*P* < 0.001, Fig. 2G). It was not surprising that the depletion of DPP3 significantly inhibited the migration ability of HCT 116 and RKO cells. Meanwhile, another shRNA targeting DDP3 in HCT 116 and RKO cells further confirmed that reduced DPP3 expression inhibited proliferation, impeded migration, and promoted apoptosis (Fig. 3A–D). Therefore, the downregulation of DPP3 contributed to the progression of CRC cells by inhibiting proliferation and migration, promoting apoptosis, and repression cycle.

### Depletion of DPP3 suppresses tumor growth in vivo

RKO cells with or without DPP3 knockdown were injected subcutaneously into mice and successfully constructed animal models for DPP3 functional validation in vivo. The bioluminescence imaging results showed that the tumor burden was reduced in shDPP3 group and the bioluminescence intensity was also obviously decreased (Fig. 3A). Furthermore, the small volume and light weight of solid tumors also suggested that tumor growth slowed down after DPP3 silencing (Fig. 3B). Apparently, according to the picture of the tumors taken out from the mice, the shDPP3 group was significantly smaller than the control group. The lower positive cells stained by Ki67 were detected in the tumor sections of shDPP3 group, which further verified the rationality of the above observation (Fig. 3C). Collectively, downregulation of DPP3 inhibited CRC tumor growth in vivo, which was consistent with the above results in vitro.

### DPP3 is involved in the regulation of CRC through CDK1

**Fig. 4:**
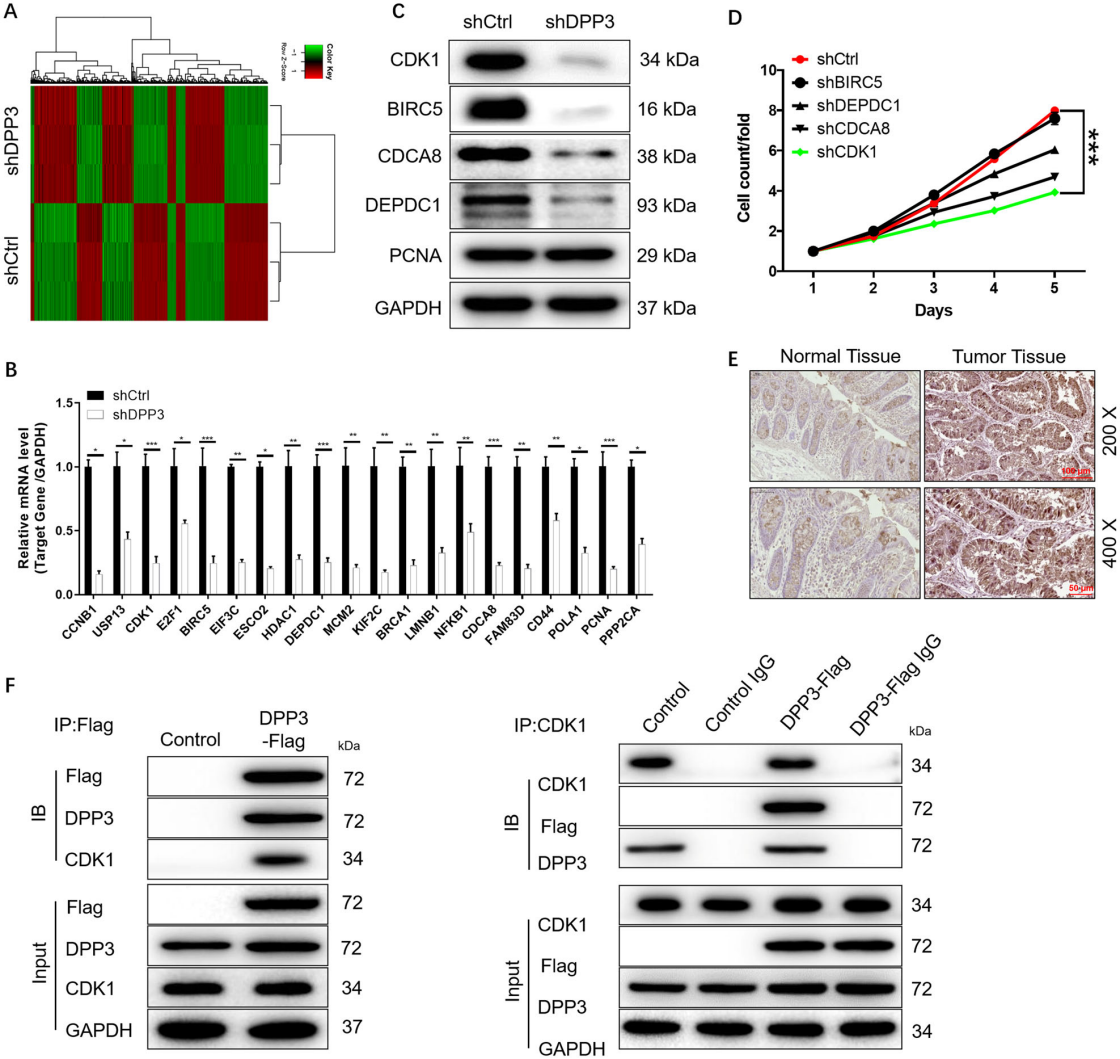
The exploration and verification of downstream underlying DPP3 induced regulation of CRC. **A** A Prime View Human Gene Expression Array was performed to identify the differentially expressed genes (DEGs) between shDPP3 and shCtrl groups of HCT 116 cells. **B**, **C** The mRNA expression of several selected DEGs in HCT 116 cells with or without DPP3 knockdown was detected by qPCR and WB (**C**). **D** Celigo cell counting assay was used to compare the effects of different targets on cell proliferation. **E** The expression of CDK1 in CRC tissues and normal tissues was evaluated by IHC analysis. **F** The potential mechanism between DDP3 and CDK1 was demonstrated through Co-IP assay. Data were shown as mean ± SD. *P < 0.05, **P < 0.01, ***P < 0.001.

Since the regulatory role of DPP3 in CRC has been basically determined, its potential mechanism was still worth further exploration. First, RNA-seq was used to identify differentially expressed genes (DEGs) in the HCT cells with or without DPP3 knockdown. According to the threshold of simultaneous |Fold Change| ≥ 1.5 and FDR < 0.05, 1704 DEGs were upregulated and 1631 DEGs were downregulated in shDPP3 compared with shCtrl (Fig. S4A, Fig. 4A). Moreover, the enrichment of 3335 DEGs in typical signaling pathways or IPA diseases and functions was evaluated functions, suggesting that the ‘cyclins and cell cycle regulation’ was one of the most abundant signaling pathways and cell cycle was one of the most abundant functions (Fig. S4B, C). Subsequently, several DEGs with high expression multiple changes in HCT 116 cells were confirmed again by qPCR (Fig. 4B) and WB (Fig. 4C). In view of the significant downregulation of these four genes (BIRC5, DEPDC1, CDCA8, and CDK1), which was further screened to identify downstream target of DPP3-mediated regulation of CRC progression. Subsequently, MTT assay screening in which lentivirus was used to deliver shRNAs into HCT 116 cells in culture indicated that the ability of cell proliferation was inhibited most significantly in shCDK1 group compared with other experimental groups (Revised Fig. 4D). Moreover, combined with the comprehensive analysis of DPP3 downstream network (Fig. S4D), CDK1 was regarded as the main downstream target of DPP3. Meanwhile, the expression of CDK1 was higher in CRC tissues than that in normal tissues (Fig. 4E). In fact, CDK1 in HCT 116 cells was also abundant (Fig. S5A). Importantly, the potential mechanism between DDP3 and CDK1 was demonstrated through Co-IP assay (Fig. 4F), suggesting that DDP3 interacted with CDK1. Depletion of DPP3 led to downregulation of CDK1, and likewise depletion of CDK1 downregulated DPP3. Accordingly, CDK1 was considered as a potential target of DPP3 in the regulation of CRC.

### Depletion of DPP3 aggravated the inhibition of CRC by CDK1 depletion

**Fig. 5:**
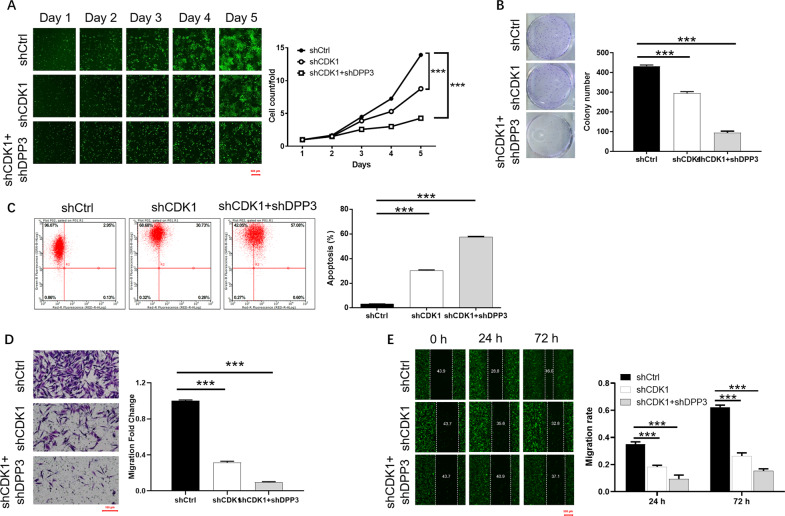
Knockdown of DPP3 deepens the effects on CRC cells by CDK1 knockdown. Cell models were subjected to the detection of cell proliferation by Celigo cell counting assay (**A**), colony formation (**B**), cell apoptosis (**C**). Cell migration was detected by Transwell assay (**D**) and wound-healing assay (**E**). The representative images were selected from at least three independent experiments. Data were shown as mean ± SD. *P < 0.05, **P < 0.01, ***P < 0.001.

HCT 116 cells with depletion of CDK1 (shCDK1) and CDK1 + DPP3 (shCDK1 + shDPP3) were established to reveal their combined effects on CRC. Like the knockdown of DPP3, the most effective CDK1 silencing shRNA was screened by qPCR (Fig. S5B). The transfection efficiency and knockdown efficiency of CDK1 were evaluated by the green fluorescence signal (Fig. S5C), qPCR (Fig. S5D), and WB (Fig. S5E), respectively. Moreover, CDK1 + NC-shDPP3, shDPP3+NC-CDK1, and CDK1 + shDPP3 in HCT 116 cells were constructed (Fig. S5F–G). Subsequent cell function experiments showed that CDK1 knockdown had a strong inhibitory effect on cell proliferation (Fig. 5A) and colony formation (Fig. 5B), and a promoting effect on cell apoptosis (Fig. 5C). In addition, both Transwell assay and wound-healing assay indicated that the loss of CDK1 significantly inhibited the migration of HCT 116 cells (Fig. 5D, E). The above experimental results verified that knockdown of CDK1 has a certain inhibitory effect on the progression of CRC, like DPP3. Furthermore, we found that the effects of DPP3 on cell proliferation, colony formation, apoptosis, and migration were aggravated in the HCT 116 cells with downregulation CDK1 and DPP3.

### Overexpression of CDK1 alleviates the inhibitory effects of DPP3 knockdown in CRC cells

**Fig. 6:**
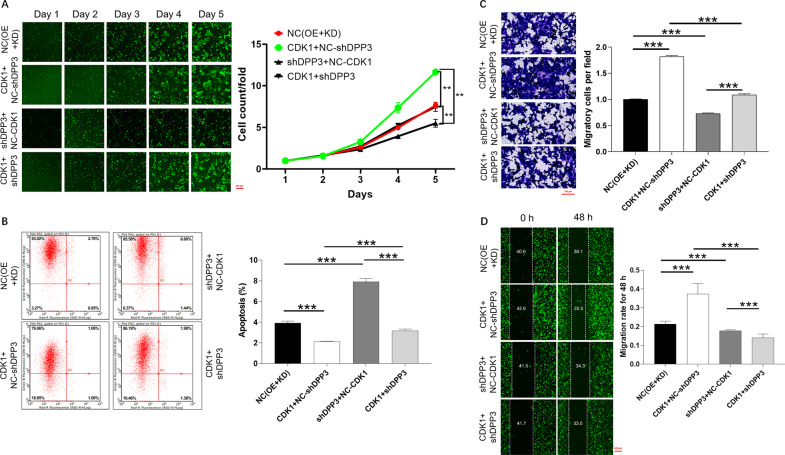
Overexpression of CDK1 alleviates the inhibitory effects of DPP3 knockdown in CRC cells. Cell models were subjected to the detection of cell proliferation (**A**), cell apoptosis (**B**). The cell migration ability was accessed by wound-healing assay (**C**) and Transwell assay (**D**). The representative images were selected from at least three independent experiments. Data were shown as mean ± SD. *P < 0.05, **P < 0.01, ***P < 0.001.

Furthermore, the results of Celigo cell count showed that compared with the control group, the cell proliferation rate in the CDK1 + NC-shDPP3 group was the strongest, followed by the CDK1 + shDPP3 group, while the cell proliferation rate in the shDPP3 +NC-CDK1 group was significantly reduced (Fig. 6A). Secondly, CDK1 + NC-shDPP3 group had the most significant inhibitory effect on apoptosis, while shDPP3 +NC-CDK1 group significantly promoted apoptosis. The apoptosis rate of CDK1 + shDPP3 group was higher than that of CDK1 + NC-shDPP3 group (Fig. 6B). Not surprisingly, cell migration in CDK1 + shDPP3 group was decreased significantly compared with other groups (Fig. 6C, D). Collectively, CDK1 overexpression can reduce the inhibitory effect of downregulation of DPP3 on CRC cells. Taken together, DPP3/CDK1 axis may exert a role in promoting the development and progression of CRC.

## Discussion

DPP3 is considered as an important regulator in substantial cellular processes. In addition, previous reports have indicated that DPP3 is related to a variety of cancers. In the present study, the role and potential molecular mechanism of DPP3 in CRC were identified for the first time. Specifically, the expression of DPP3 was positively associated with lymphatic metastasis, pathological stage, positive number of lymph nodes in CRC patients. The results of Kaplan–Meier survival analysis confirmed that high DPP3 expression predicted poor prognosis in CRC patients. In addition, reduced expression of DPP3 significantly inhibited cell proliferation and migration, induced apoptosis, and arrested cell cycle in G2 phase of CRC cells in vitro. Unsurprisingly, the cells with downregulated DPP3 expression were less tumorigenic, and the tumors growth rate was relatively slow in vivo. All the results elucidated that DPP3 was involved in progression and development of CRC as well as had oncogene-like characteristics.

Apoptosis is an important mechanism for all multicellular organisms to control cell proliferation, maintain tissue dynamic balance, and eliminate harmful or unnecessary cells^18^. It is important to identify the mechanisms of apoptosis to understand the progression and development of the disease. BID, a pro-apoptotic member of the Bcl-2 family, was found to be directly regulated by miR-20a in CRC cells, thus inducing mitochondrial apoptosis^19^. BIM and SMAC mediated intrinsic apoptosis regulates the apoptosis of CRC cells^20,21^. In addition, HSP60 may play a role in promoting cell death by enhancing pro-Caspase3 or increasing protein ubiquitination^22^. The process further activated the protease family of downstream (Caspases), which leads to apoptosis^23,24^. Moreover, cell cycle inhibitors p21, p27, and transcription factor p53 were all tumor suppressor, which can induce growth arrest or apoptosis of cancer cells^25–28^. On the other hand, CRC is among the most common lethal types of cancer worldwide, which resistant to conventional chemotherapy through high expression of anti-apoptotic proteins^29^. The development of molecular targets for apoptosis induction may be beneficial to patients with CRC^30^. In this study, we found that the depletion of DPP3 could promote apoptosis of CRC cells by upregulating the pro-apoptotic expression, such as BID, BIM, SMAC, HSP60, Caspase3, Caspase8, p21, p27, and p53. Accordingly, inhibition of DPP3 expression may be a potential anticancer target for patients with CRC.

Cell cycle progression is the core event of all proliferating cells, which is mainly driven by cyclin-dependent kinase (CDKs)^31^. Cyclin-dependent kinase 1 (CDK1) is the only necessary CDK that facilitates the G2-M transition and regulates G1 progression and G1-S transition^32,33^. Recent studies have shown that CDK1 not only regulates the cell cycle, but also plays an important role in the proliferation of tumor cells. In particular, accumulation of cytoplasmic CDK1 is associated with cancer growth and survival rate in epithelial ovarian cancer^34^. Furthermore, CDK1 was frequently overexpressed in hepatocellular carcinoma and associated with tumor progression^35^.

Zhang et al., clarified that CDK1 is overexpressed in CRC cells and is sensitive to apoptosis^36^. Similarly, this study found that CDK1 was overexpressed in CRC tissues and cell lines. Meanwhile, downregulation of CDK1 inhibited the proliferation, colony formation, and cell migration as well as promoted apoptosis in CRC cells. Interestingly, downregulation of DPP3 may lead to downregulation of CDK1, and vice versa, suggesting that DPP3 interacts with CDK1. Simultaneously, depletion of DPP3 and CDK1 aggravated the inhibition effects than mere CDK1 knockdown in CRC.

In summary, DPP3 is involved in the regulation of the progression and development of CRC by targeting CDK1, which is a potential prognostic and therapeutic target of CRC.

## Supplementary information

Supplementary figure legends

Table S1

Table S2

Figure S1

Figure S2

Figure S3

Figure S4

Figure S5

HCT116 STR profiling

RKO STR profiling
