# Correction: DPP3/CDK1 contributes to the progression of colorectal cancer through regulating cell proliferation, cell apoptosis, and cell migration

**DOI:** 10.1038/s41419-021-03886-3

**Published:** 2021-06-16

**Authors:** Yixin Tong, Yuan Huang, Yuchao Zhang, Xiangtai Zeng, Mei Yan, Zhongsheng Xia, Dongming Lai

**Affiliations:** 1 Department of Gastrointestinal Surgery, Sun Yat-Sen Memorial Hospital of Sun Yat-Sen University, 107 Yanjiang West Road, Guangzhou, Guangdong Province China; 2 Department of Tongji Medical College of Huazhong University, 1095 Jiefang Dadao, Wuhan, Hubei Province China; 3 Department of Endoscopy Center, Zhongshan Hospital of Fudan University, 180 Fenglin Road, Xuhui, Shanghai China; 4 Department of The First Affiliated Hospital, Gannan Medical University, 23 Qingnian Road, Zhanggong District, Ganzhou, Jiangxi Province China

**Keywords:** Cancer, Diseases

Correction to: *Cell Death and Disease*

10.1038/s41419-021-03796-4 published online 22 May 2021

The original version of this article unfortunately contained a mistake in the affiliations. We apologize for the error. The original article has been corrected. The correct affiliations are: Yixin Tong^1^, Yuan Huang^2^, Yuchao Zhang^3^, Xiangtai Zeng^4^, Mei Yan^3^, Zhongsheng Xia^3^, Dongming Lai^3^

^1^Department of Tongji Medical College of Huazhong University, 1095 Jiefang Dadao, Wuhan, Hubei Province, China.

^2^Department of Endoscopy Center, Zhongshan Hospital of Fudan University, 180 Fenglin Road, Xuhui, Shanghai, China.

^3^Department of Gastrointestinal Surgery, Sun Yat-Sen Memorial Hospital of Sun Yat-Sen University, 107 Yanjiang West Road, Guangzhou, Guangdong Province, China.

^4^Department of The First Affiliated Hospital, Gannan Medical University, 23 Qingnian Road, Zhanggong District, Ganzhou, Jiangxi Province, China.

