# Supplementary material for: DPP3/CDK1 contributes to the progression of colorectal cancer through regulating cell proliferation, cell apoptosis, and cell migration
Source: Cell Death Dis. 2021 May 22;12(6):529. doi: 10.1038/s41419-021-03796-4 (PMC8141054; doi:10.1038/s41419-021-03796-4)
Supplement: Supplementary file 1 — Supplementary figure legends [file 41419_2021_3796_MOESM1_ESM.docx]

**Figure S1.** (A) The expression of DPP3 in CRC cell lines was accessed by qPCR. (B) The knockdown efficiencies of 3 shRNAs designed for DPP3 knockdown were evaluated by qPCR. (C) The transfection efficiencies of shDPP3 and shCtrl in RKO and HCT116 cells were evaluated through observing the fluorescence of GFP on lentivirus vector. The knockdown efficiency of DPP3 in RKO and HCT 116 cells was assessed by qPCR (D) and WB (E). Data was shown as mean ± SD. **P* < 0.05, ***P* < 0.01

**Figure S2.** Another shRNA targeting DDP3 cell was successfully constructed and subjected to the detection of transfection efficiency (A) and knockdown efficiency (B, C) in RKO and HCT 116 cells. Data was shown as mean ± SD. *P < 0.05, **P < 0.01

**Figure S3.** shRNA targeting DDP3 cell was successfully constructed and subjected to the detection of proliferation (A), cell apoptosis (B) and migration (C, D). The representative images were selected from at least 3 independent experiments. Data was shown as mean ± SD. *P < 0.05, **P < 0.01, ***P < 0.001

**Figure S4.** (A) The volcano plot of gene expression profiling in HCT116 cells with or without DPP3 knockdown. Red dots represented significantly upregulated DEGs. Blue dots represented significantly downregulated DEGs. (B) The enrichment of the DEGs in canonical signaling pathways was analyzed by IPA. (C) The enrichment of the DEGs in IPA disease and function was analyzed by IPA. (D) All bioinformatics and IPA DPP3-related interaction networks. Data was shown as mean ± SD. *P < 0.05, **P < 0.01, ***P < 0.001

**Figure S5.** (A) The expression of CDK1 in CRC cell lines was accessed by qPCR. (B) The knockdown efficiencies of 3 shRNAs designed for CDK1 knockdown were evaluated by qPCR. (C) The transfection efficiencies of shCtrl, shCDK1, shCDK1+ shDPP3 in HCT 116 cells were evaluated through observing the fluorescence of GFP on lentivirus vector. (D, E) The knockdown efficiencies of CDK1 were evaluated by qPCR (D) and WB (E). (F) The transfection efficiency of NC(OE+KD), CDK1+NC-shDPP3, shDPP3+NC-CDK1 and CDK1+shDPP3 in HCT116 cells were evaluated through observing the fluorescence of GFP on lentivirus vector. (G) The protein expression of CDK1 and DPP3 of NC(OE+KD), CDK1+NC-shDPP3, shDPP3+NC-CDK1 and CDK1+shDPP3 was evaluated in HCT116cells by WB. Data was shown as mean ± SD. ****P* < 0.001
