# Supplementary material for: DPP3/CDK1 contributes to the progression of colorectal cancer through regulating cell proliferation, cell apoptosis, and cell migration
Source: Cell Death Dis. 2021 May 22;12(6):529. doi: 10.1038/s41419-021-03796-4 (PMC8141054; doi:10.1038/s41419-021-03796-4)
Supplement: Supplementary file 2 — Table S1 [file 41419_2021_3796_MOESM2_ESM.docx]

Antibodies used in IHC

| Primary antibodies | Dilution in IHC | Source species | Company | Catalog No. |
| --- | --- | --- | --- | --- |
| Ki67 | 1:400 | Rabbit | abcam | ab6721 |
| DPP3 | 1:50 | Rabbit | Invitrogen | PA5-35038 |
| CDK1 | 1:100 | Rabbit | abcam | ab133327 |
| Secondary antibody | Dilution |  | Company | Catalog No. |
| HRP Goat Anti-Rabbit IgG | 1:200 |  | Abcam | Ab111909 |

Antibodies used in WB

| Antibody Name | Band Size (KDa) | Diluted Multiples | Antibody Source | Company | Number |
| --- | --- | --- | --- | --- | --- |
| DPP3 | 83 | 1:2000 | Rabbit | Invitrogen | PA5-35038 |
| CDK1 | 34 | 1:1500 | Rabbit | abcam | ab133327 |
| Akt | 60 | 1:1000 | Rabbit | CST | 4685 |
| p-Akt | 60 | 1:1000 | Rabbit | Bioss | BS-5193R |
| CCND1 | 36 | 1:2000 | Rabbit | CST | 2978 |
| CDK6 | 37 | 1:1000 | Rabbit | abcam | ab151247 |
| PIK3CA | 110 | 1:1000 | Rabbit | abcam | ab40776 |
| GAPDH | 37 | 1:3000 | Rabbit | Bioworld | AP0063 |

| Secondary antibody | Dilution |  | Company | Catalog No. |
| --- | --- | --- | --- | --- |
| HRP Goat Anti-Rabbit IgG | 1:3000 |  | Beyotime | A0208 |

Antibodies used in Co-IP

| Antibody Name | Band Size (KDa) | Diluted Multiples | Antibody Source | Company | Number |
| --- | --- | --- | --- | --- | --- |
| DPP3 | 83 | 1:1000 | Rabbit | bioss | bs-13029R |
| CDK1 | 34 | 1:10/1:1000 | Rabbit | abcam | ab133327 |
| DYKDDDK Tag |  | 1:50/1:1000 | Rabbit | CST | 14793 |
| GAPDH | 37 | 1:3000 | Rabbit | Bioworld | AP0063 |

| Secondary antibody | Dilution |  | Company | Catalog No. |
| --- | --- | --- | --- | --- |
| HRP Goat Anti-Rabbit IgG | 1:3000 |  | Beyotime | A0208 |
