# Supplementary material for: DPP3/CDK1 contributes to the progression of colorectal cancer through regulating cell proliferation, cell apoptosis, and cell migration
Source: Cell Death Dis. 2021 May 22;12(6):529. doi: 10.1038/s41419-021-03796-4 (PMC8141054; doi:10.1038/s41419-021-03796-4)
Supplement: Supplementary file 3 — Table S2 [file 41419_2021_3796_MOESM3_ESM.docx]

The target sequences and shRNA sequences

| Target | Serial Number | Target Sequence (5’-3’) |
| --- | --- | --- |
| Human-DPP3-1 | Pbr15670 | CTTCAAGAGGTCGATGGAGA |
| Human-DPP3-2 | Pbr15671 | CCGAGGAGAATTTGAAGGTTT |
| Human-DPP3-3 | Pbr15672 | GCTGGAGAAAGCCAAGGCCTA |
| Human-CDK1-1 | Pbr10545 | TTCCATGGATCTGAAGAAATA |
| Human-CDK1-2 | Pbr10546 | AGACTAGAAAGTGAAGAGGAA |
| Human-CDK1-3 | Pbr10547 | ATGGAGTTGTGTATAAGGGTA |

Primers used in qPCR

| Primer Name | Upstream Primer  Sequence (5’-3’) | Downstream Primer  Sequence (5’-3’) |
| --- | --- | --- |
| DPP3 | TGAGTGCCAAGTTTGAGCG | AGCGAAGGTGAGAACATCCAG |
| CDK1 | CCATACCCATTGACTAACTAT | ACCCCTTCCTCTTCACTTTC |
| GAPDH | TGACTTCAACAGCGACACCCA | CACCCTGTTGCTGTAGCCAAA |
