# Supplementary figures and images for: DPP3/CDK1 contributes to the progression of colorectal cancer through regulating cell proliferation, cell apoptosis, and cell migration
Source: Cell Death Dis. 2021 May 22;12(6):529. doi: 10.1038/s41419-021-03796-4 (PMC8141054; doi:10.1038/s41419-021-03796-4)

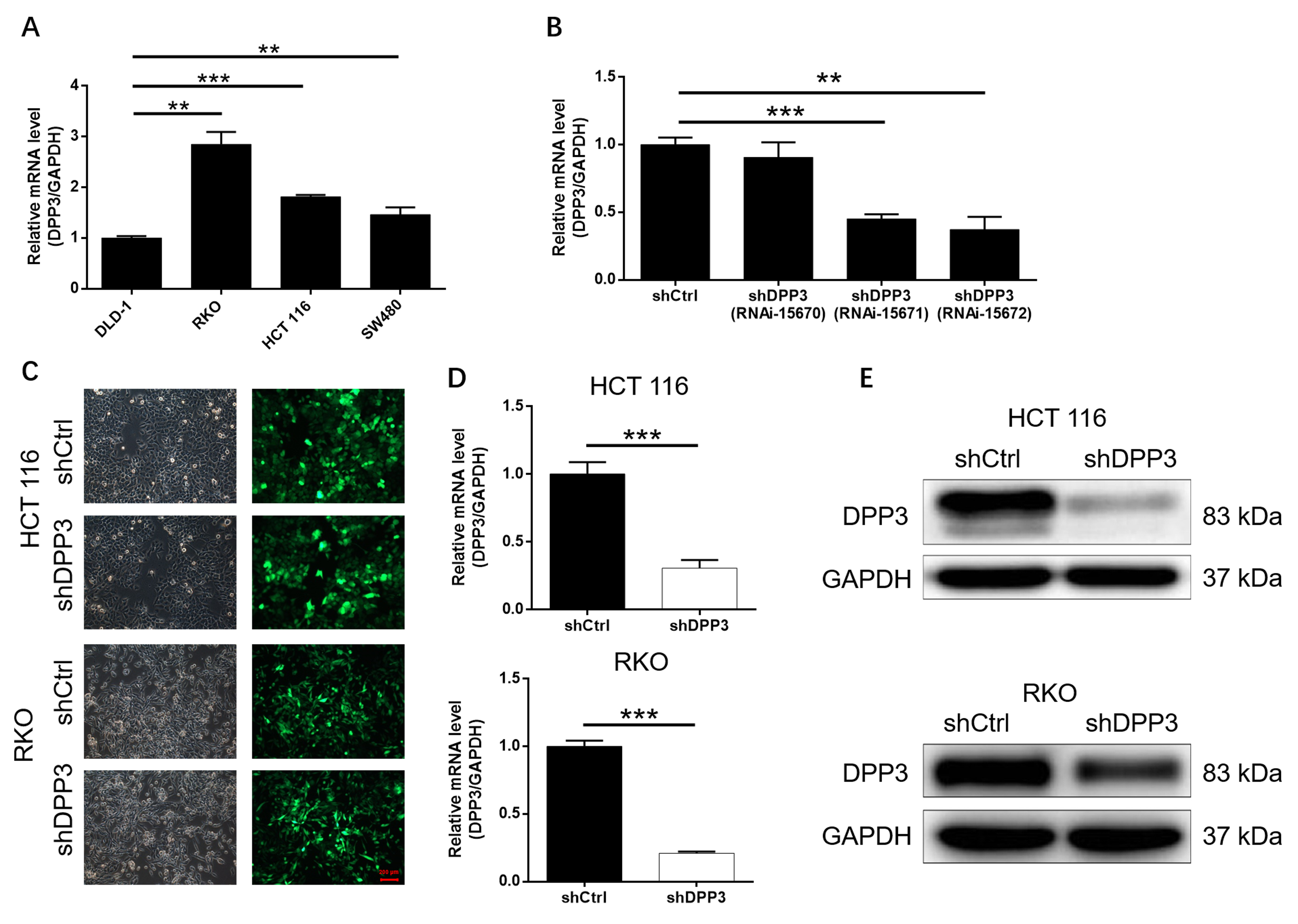

Supplement: Supplementary file 4 — Figure S1 [file 41419_2021_3796_MOESM4_ESM.tif]

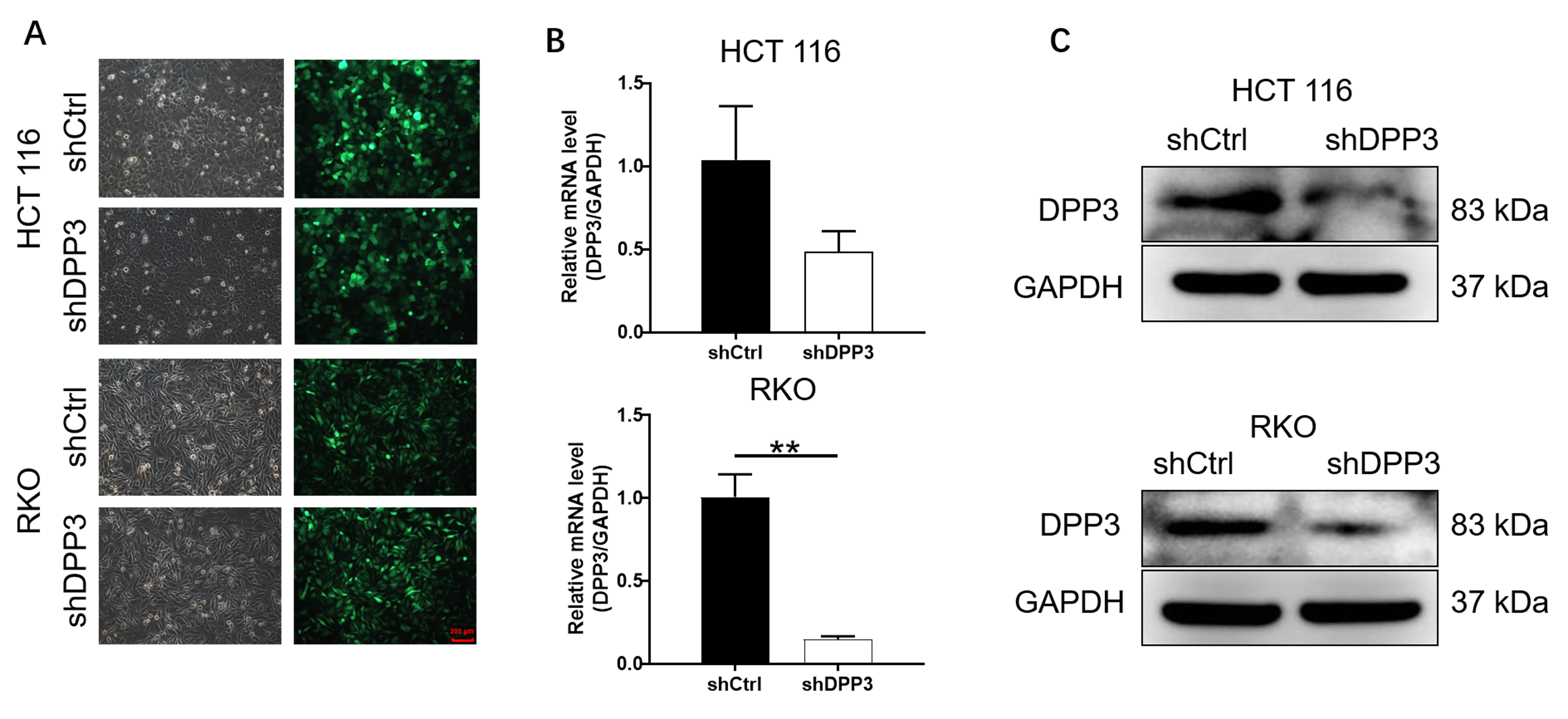

Supplement: Supplementary file 5 — Figure S2 [file 41419_2021_3796_MOESM5_ESM.tif]

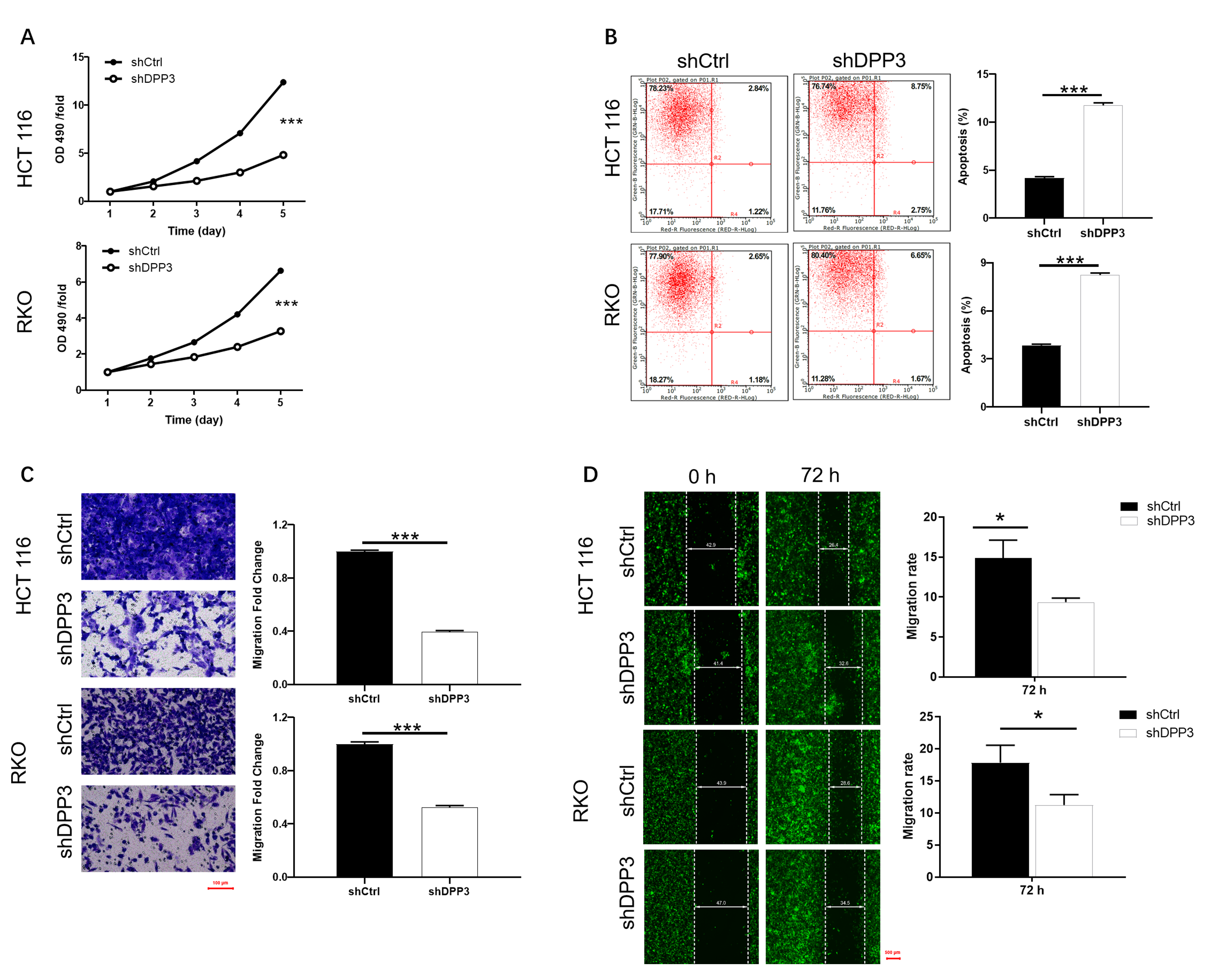

Supplement: Supplementary file 6 — Figure S3 [file 41419_2021_3796_MOESM6_ESM.tif]

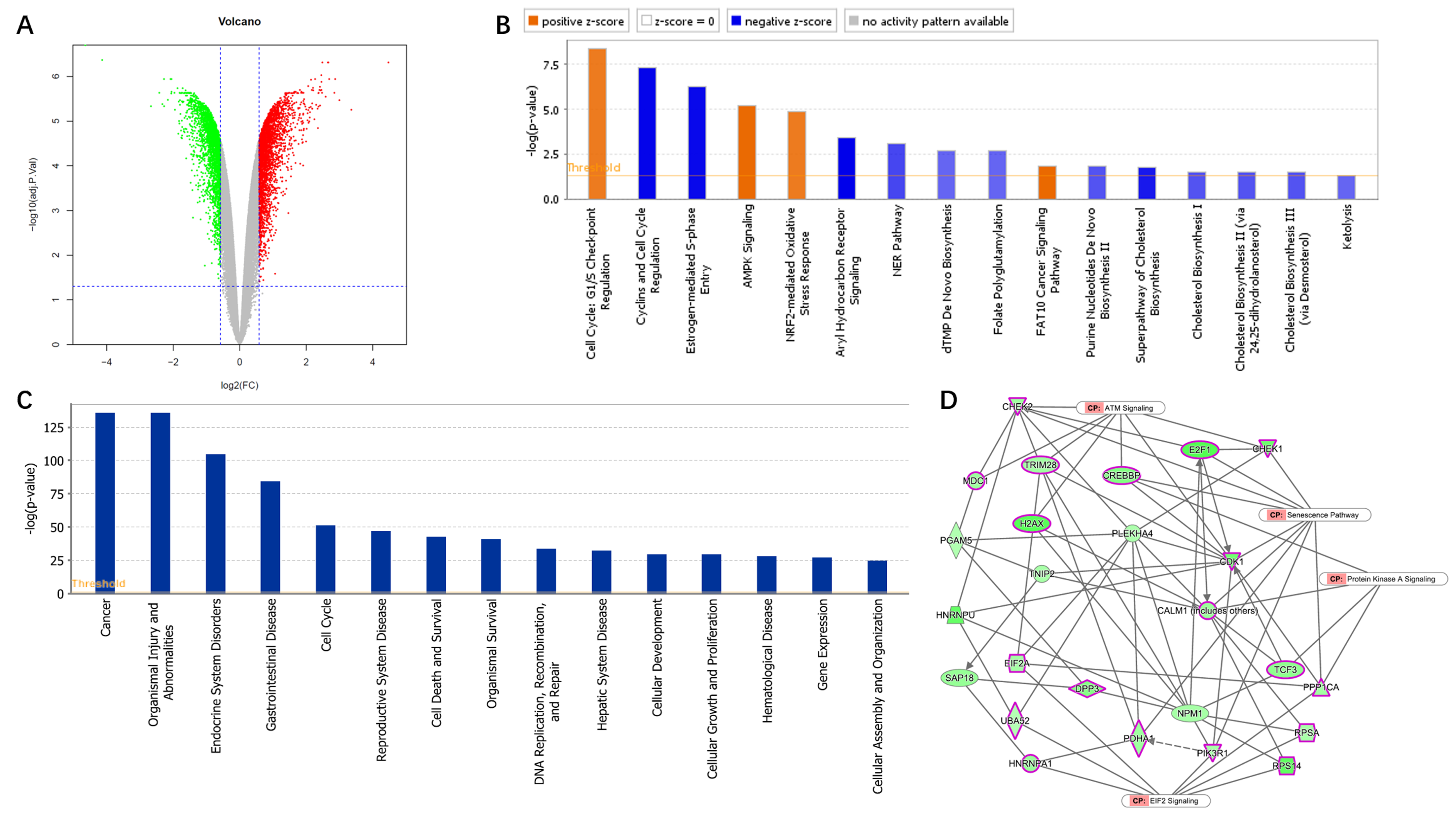

Supplement: Supplementary file 7 — Figure S4 [file 41419_2021_3796_MOESM7_ESM.tif]

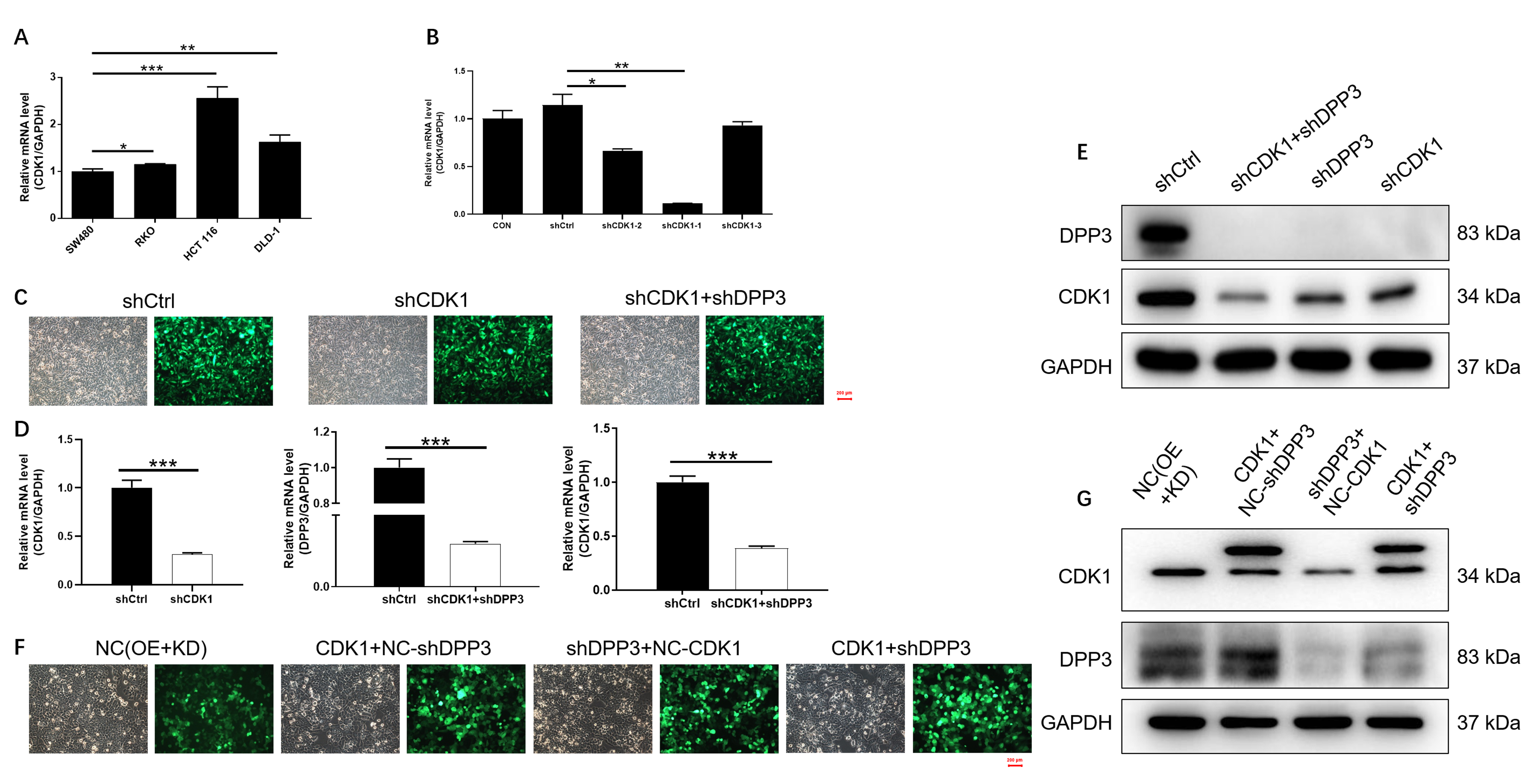

Supplement: Supplementary file 8 — Figure S5 [file 41419_2021_3796_MOESM8_ESM.tif]

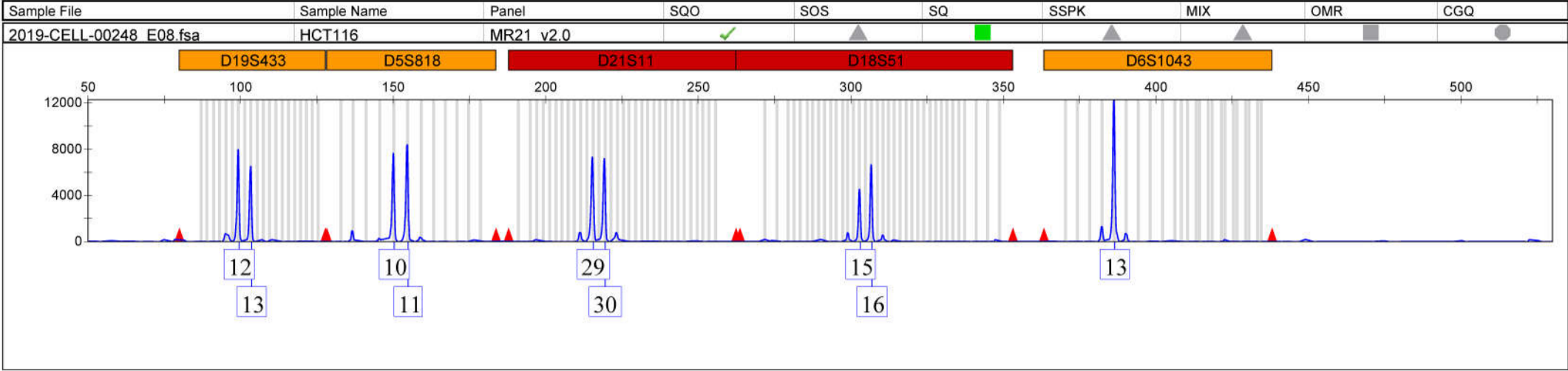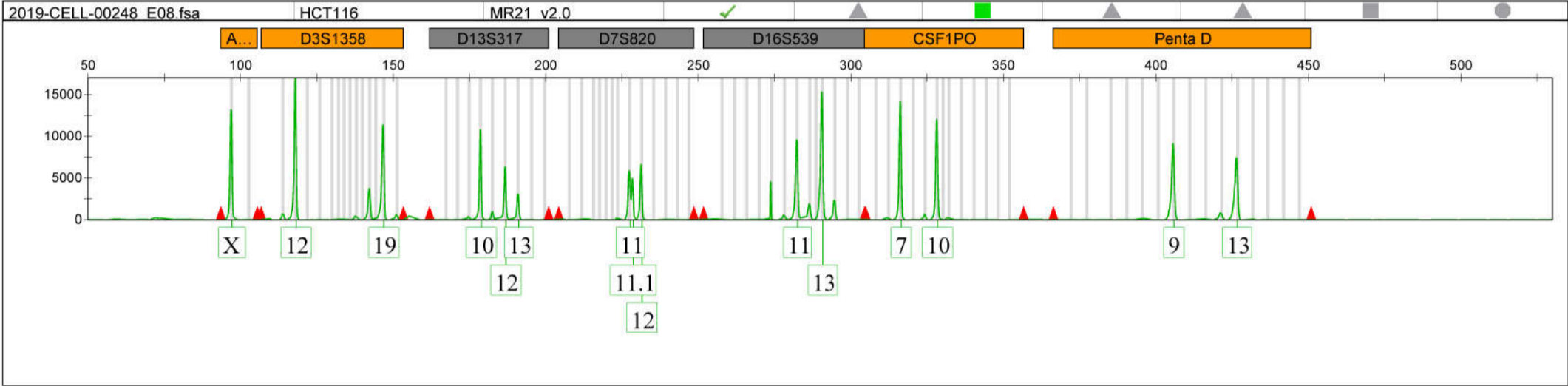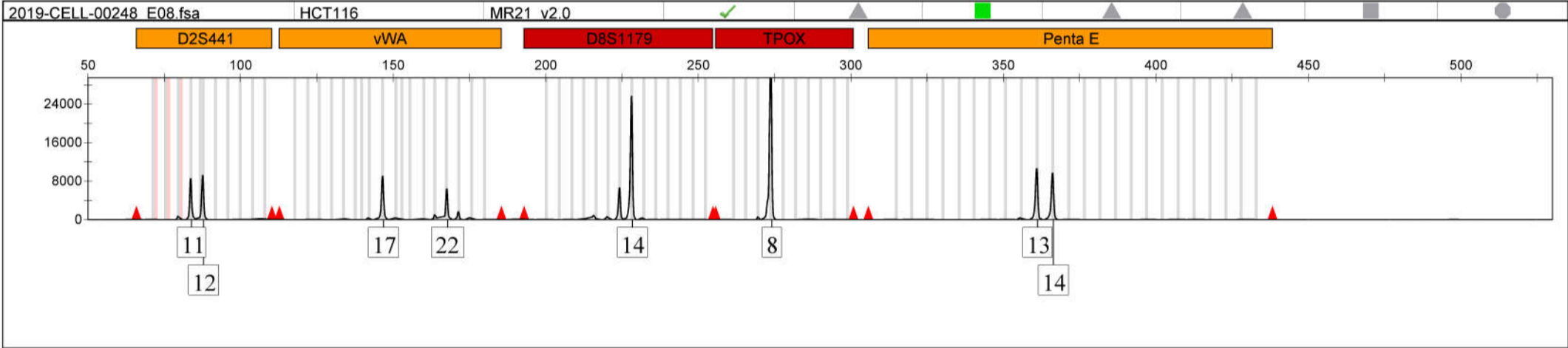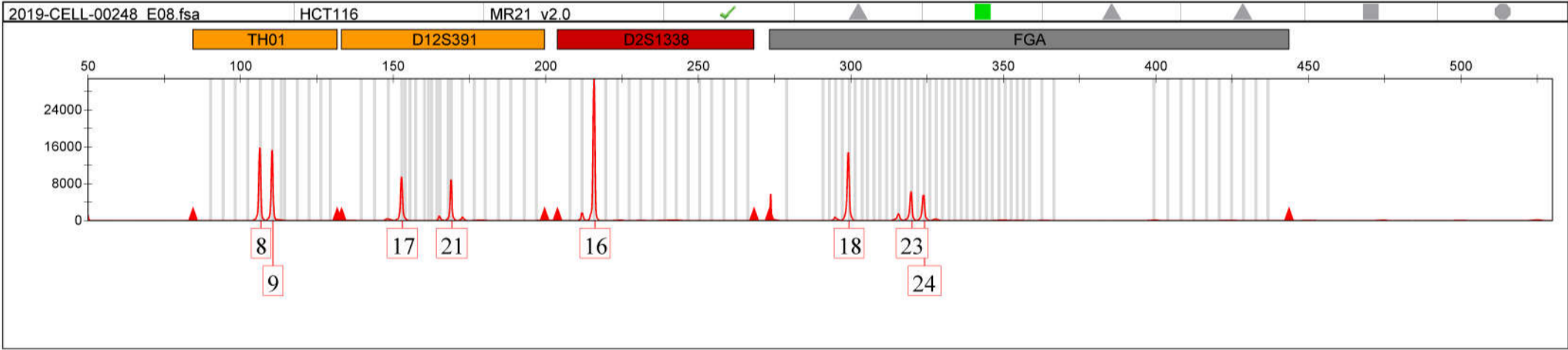

| EV          | Cell No. | Cell name | Locus names |          |            |         |       |      |     |      |        | Figures |
|-------------|----------|-----------|-------------|----------|------------|---------|-------|------|-----|------|--------|---------|
|             |          |           | D5S818      | D13S317  | D7S820     | D16S539 | VWA   | TH01 | AM  | TPOX | CSF1PO |         |
|             |          |           | 10,11       | 10,12,13 | 11,11.1,12 | 11,13   | 17,22 | 8,9  | x,x | 8,8  | 7,10   |         |
| 0.84(32/38) | CCL-247  | HCT 116   | 10,11       | 10,12    | 11,12      | 11,13   | 17,22 | 8,9  | X,Y | 8,9  | 7,10   | -       |

Supplement: Supplementary file 9 — HCT116 STR profiling [file 41419_2021_3796_MOESM9_ESM.pdf]

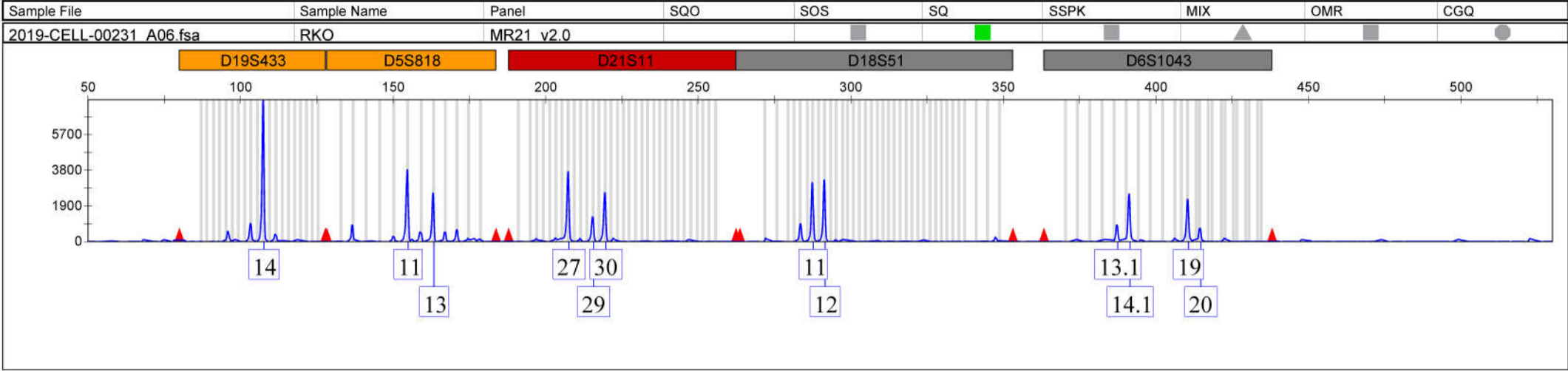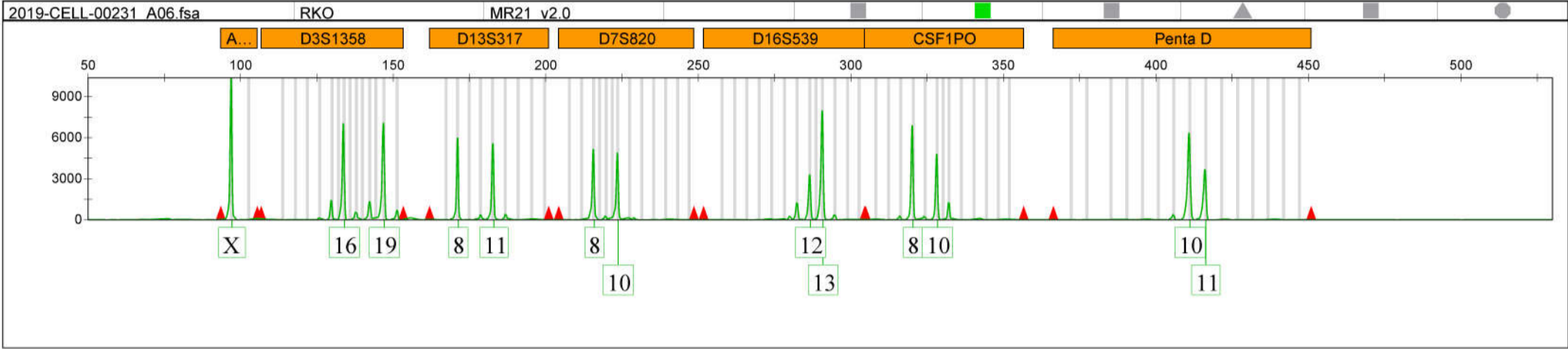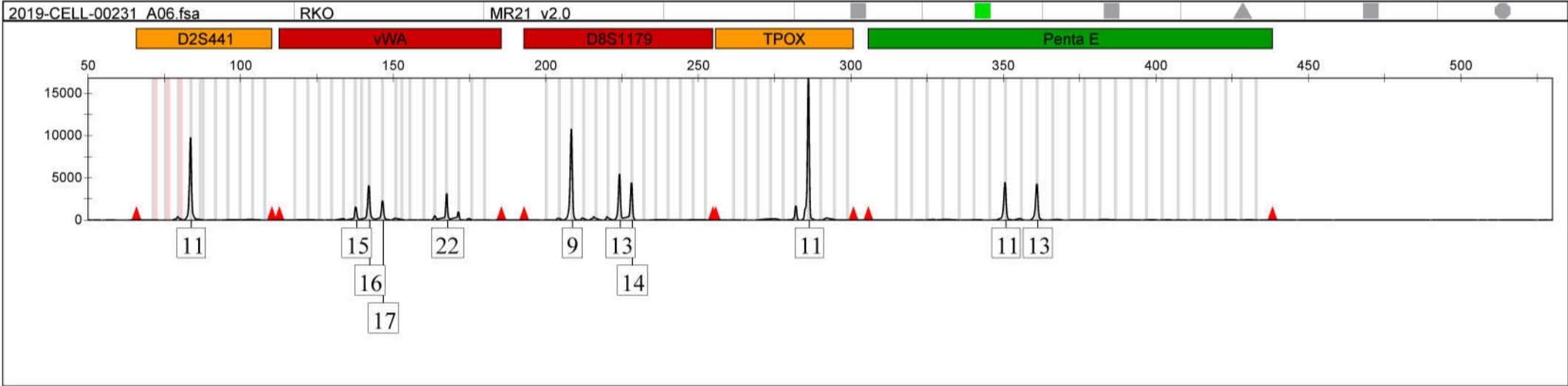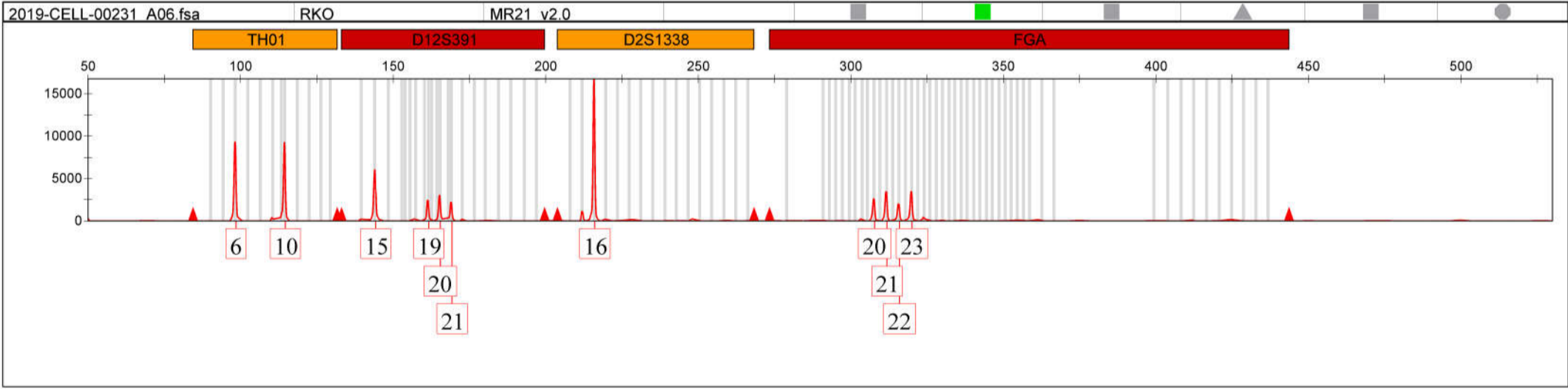

| EV          | Cell No. | Cell name | Locus names |         |        |         |             |      |     |       |        | Figures |
|-------------|----------|-----------|-------------|---------|--------|---------|-------------|------|-----|-------|--------|---------|
|             |          |           | D5S818      | D13S317 | D7S820 | D16S539 | VWA         | TH01 | AM  | TPOX  | CSF1PO |         |
|             |          |           | 11,13       | 8,11    | 8,10   | 12,13   | 15,16,17,22 | 6,10 | X,X | 11,11 | 8,10   |         |
| 0.97(38/39) | CRL-2577 | RKO       | 11,13       | 8,11    | 8,10   | 12,13   | 15,16,17    | 6,10 | X,X | 11,11 | 8,10   | -       |

Supplement: Supplementary file 10 — RKO STR profiling [file 41419_2021_3796_MOESM10_ESM.pdf]
